# Small interfering RNAs in the management of human rheumatoid arthritis

**DOI:** 10.1093/bmb/ldac012

**Published:** 2022-04-29

**Authors:** Giuseppe Gargano, Francesco Oliva, Antonio Oliviero, Nicola Maffulli

**Affiliations:** Department of Trauma and Orthopaedic Surgery, AOU San Giovanni di Dio e Ruggi D’Aragona, Via San Leonardo 1, Salerno 84131, Italy; Department of Medicine, Surgery and Dentistry, University of Salerno, Via S. Allende, Baronissi SA 84081, Italy; Department of Trauma and Orthopaedic Surgery, AOU San Giovanni di Dio e Ruggi D’Aragona, Via San Leonardo 1, Salerno 84131, Italy; Department of Medicine, Surgery and Dentistry, University of Salerno, Via S. Allende, Baronissi SA 84081, Italy; Department of Trauma and Orthopaedic Surgery, AOU San Giovanni di Dio e Ruggi D’Aragona, Via San Leonardo 1, Salerno 84131, Italy; Department of Medicine, Surgery and Dentistry, University of Salerno, Via S. Allende, Baronissi SA 84081, Italy; Department of Trauma and Orthopaedic Surgery, AOU San Giovanni di Dio e Ruggi D’Aragona, Via San Leonardo 1, Salerno 84131, Italy; Department of Medicine, Surgery and Dentistry, University of Salerno, Via S. Allende, Baronissi SA 84081, Italy; Queen Mary University of London, Barts and the London School of Medicine and Dentistry, Centre for Sports and Exercise Medicine, Mile End Hospital, 275 Bancroft Road, London E1 4DG, UK; School of Pharmacy and Bioengineering, Keele University School of Medicine, Thornburrow Drive, Stoke on Trent, ST4 7QB, UK

**Keywords:** rheumatoid arthritis, rheumatoid arthritis therapy, small interfering RNA, short interfering RNA, silencing RNA, RNA interference

## Abstract

**Background:**

Rheumatoid arthritis (RA) has unclear pathogenesis, but the molecules that feed its inflammatory state are known. Small interfering RNAs (siRNAs) are useful to identify molecular targets and evaluate the efficacy of specific drugs, and can themselves be used for therapeutic purposes.

**Sources of data:**

A systematic search of different databases to March 2022 was performed to define the role of siRNAs in RA therapy. Twenty suitable studies were identified.

**Areas of agreement:**

Small interfering RNAs can be useful in the study of inflammatory processes in RA, and identify possible therapeutic targets and drug therapies.

**Areas of controversy:**

Many genes and cytokines participate in the inflammatory process of RA and can be regulated with siRNA. However, it is difficult to determine whether the responses to siRNAs and other drugs studied in human cells *in vitro* are similar to the responses *in vivo*.

**Growing points:**

Inflammatory processes can be affected by the gene dysregulation of siRNAs on inflammatory cytokines.

**Areas timely for developing research:**

To date, it is not possible to determine whether the pharmacological response of siRNAs on cells *in vitro* would be similar to what takes place *in vivo* for the diseases studied so far.

## Introduction

Rheumatoid arthritis (RA) is a chronic autoimmune disease that primarily affects the small joints.

Joints are often hot, swollen and painful. The most commonly affected sites are the wrists and hands on both sides. Other less-affected sites are skin, heart, eyes, lung, blood and nerves. Inflammation of the lungs and heart and reduced number of red blood cells can occur.^1^ Systemic symptoms may include fever, asthenia and consequent weight loss. Symptoms often evolve slowly and manifest within weeks or months. The cause of RA is not entirely clear, and it is hypothesized that it is a multifactorial mechanism that includes both genetic and environmental factors.^2^ The pathological mechanism is determined by an autoimmune reaction, where the immune system attacks the joints. There is inflammation and thickening of the joint capsule with involvement of the bones and underlying cartilage.^3^ Although the diagnosis is clinical, laboratory tests and radiographs are used to confirm it and rule out other conditions. To diagnose RA, it is necessary to exclude other pathologies that can present in a similar way, including psoriatic arthritis, systemic lupus erythematosus and fibromyalgia.^1^

In recent years, more attention has been paid to the natural processes of regulation of eukaryotic genes, in particular RNA interference (RNAi).^4^^,^^5^ The aim is to use this mechanism in genomic research and in the formulation of suitable therapies.^6–8^ Usually, a small interfering RNA (siRNA) is composed of about 20 nucleotides arranged to form a double-stranded RNA molecule.^9^

The RNAi mechanism involves various elements such as detection wire (passenger wire), sense wire (guide wire), enzymes such as dicer, argonaute and the central part RNA-induced silencing complex (RISC).^10^ The guide wire is a nucleotide sequence recognized by Dicer, which selects it and integrates it into RISC. The guide wire is used to recognize the passenger wire, which will be degraded by RISC.^11^

With the study of the physiological role of siRNAs and the understanding of their mechanism of action, it is easier to find a therapeutic application that is targeted on the expression of specific genes.^12^ The field of application is varied, and includes endocrinological abnormalities, hepatitis, tumors and pathologies triggered by external agents such as viruses.^11^

Small interfering RNAs selectively target a gene and silence it, inhibiting the expression of the gene that determines the pathology under study.

To date, the sequence of 4894 chemically modified siRNAs is available.^13^

The mechanism of action of siRNA is very effective for the study of human genomics and for the development of possible therapies, but it has an intrinsic issue, as siRNAs are easily degradable by nucleases, which give them a short half-life. To improve their stability and determine a better half-life and efficacy, chemical modifications have been made.^13^ 128 unique chemical changes were analyzed at different locations with various permutations, specific combinations or additions such as the modification of fluorine to 2-OH at different nucleotide positions of siRNA to maintain silencing power and increase thermal stability.^13^

In RA, the autoimmune response and inflammatory response are mediated by numerous cytokines that result in a self-sustaining cascade reaction.

Some authors have tried to use siRNAs to block some biological signaling pathways, thereby blocking the inflammatory response.^14^^,^^15^ Other authors have instead used siRNAs to evaluate and monitor the inflammatory and autoimmune response after the administration of specific drugs.^16–19^

This review evaluates the current scientific evidence on the use of siRNAs in RA therapy.

Only published articles performed with human cells were selected.

## Methods

**Fig. 1:**
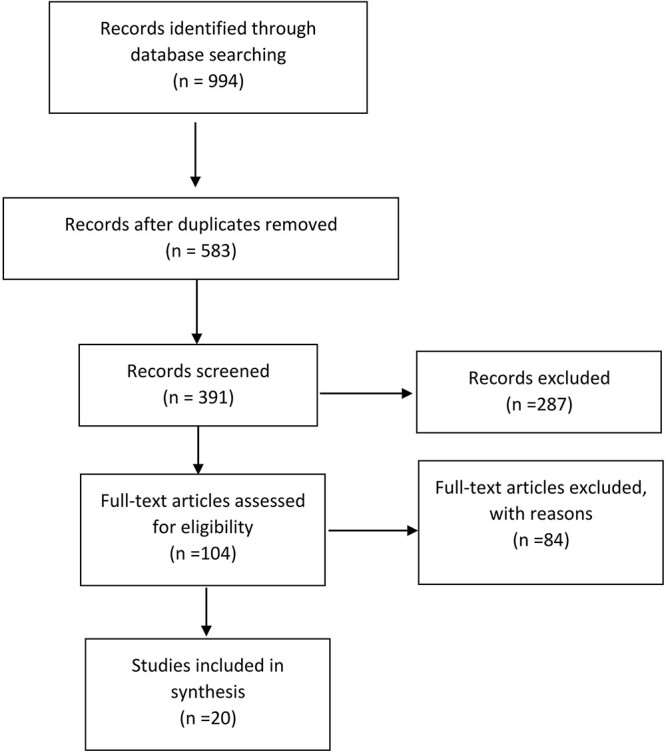
PRISMA flow diagram.

The review follows the Preferred reporting items for systematic reviews and meta-analyses (PRISMA)^20^ (Fig. 1).

All published investigations reported the possible role of siRNA in RA therapy according to *a priori* established inclusion criteria.

Studies in language other than English were included from the present investigation. Narrative and systematic reviews, meta-analyses, technical notes and case reports were excluded.

Articles in all languages were included, with no time limit for publication. All techniques of using siRNAs in RA therapy were included.

Two investigators independently conducted the systematic search, through March 2022, from the full-text archives of Embase, Google Scholar, Scopus and PubMed. In the search, we used combinations of the following key terms: RA, siRNA, short interfering RNA, silencing RNA, RNAi, with no limit of the year of publication. Two investigators independently examined the titles and abstracts to remove duplicates, and evaluated the eligible studies according to the preestablished inclusion criteria. If titles and abstracts did not allow to decide on inclusion or exclusion, the relevant full text was examined. The bibliographies of the articles included were reviewed by hand to identify further related articles. If discrepancies persisted, discussion with the senior investigator allowed to resolve them.

Twenty studies satisfied the inclusion criteria, and were thus included in the analysis. The details of the search are detailed in the flowchart in Figure 1.

## Results

A total of 994 articles were identified by the search engines used and the duplicates were subsequently removed, obtaining 583 articles. At this point, 391 articles were excluded after reading the titles and abstracts. Narrative and systematic reviews, meta-analyses, technical notes and case reports were excluded. There were 104 articles left and 84 articles were excluded as they were not appropriate for the topics covered or for the incomplete amount of information possessed.

Twenty studies met the inclusion criteria, data were extracted and collected in Table 1.

**Table 1:** Studies included

Study	Small interfering RNA target gene^*^	Function of RA therapy	Drugs activity tested	Cells analyzed	Type of study
Lee *et al*. ^21^	HIF-1*α*	HIF-1*α* is used to monitor the effect of hypoxia on the inflammatory process	HIF-1*α* – siRNA	Fibroblasts	*in vitro*
Ikuta *et al*. ^16^	Sp1	Sp1 inhibits GLS, which has angiogenic and arthritogenic activities	Mithramycin	FLS	*in vitro*
Park *et al*. ^17^	COX-2	COX-2 is implicated in the inflammatory response	Dexamethasone, COX-2-siRNA	Chondrocyte	*in vitro*
Li *et al*. ^18^	HMGB1	HMGB1 is associated with the development of RA.	MTX	FLS	*in vitro*
Municio *et al*. ^19^	TS	TS acts on p53 therefore on macrophage activity	MTX	Macrophages	*in vivo*
Xu *et al*. ^23^	Cyr61	Cyr61 acts on matrix MMP-3 and MMP-13, mediates cell adhesion, migration and cell apoptosis in inflammatory processes of RA	Cyr61-siRNA	FLS	*in vitro*
Chen *et al*. ^22^	PRMT5	PRMT5 regulate inflammation, cell proliferation, migration and invasion of FLSs	PRMT5-siRNA	FLS	*in vitro*
Nogueira *et al*. ^24^	Mcl-1	Mcl-1 is a protein essential for synovial macrophage survival	MCL1 siRNA	Macrophages	*in vitro*
Wang *et al*. ^25^	RRM2	RRM2 is a critical protein for DNA synthesis and repair, which promotes the proliferation of cells and inhibits cellular apoptosis.	RRM2 siRNA	FLS	*in vitro*
Li *et al*. ^33^	STAT3	STAT3 modulates the signaling of Th17 transcription, transduction and activation present in the RA	As2O3	Th17	*in vitro*
Ma *et al*. ^34^	PDK-1	PDK-1 stimulates invasion and migration of FLS from RA patients	Artesunate	FLS	*in vitro*
Peng *et al*. ^14^	COX-2	COX-2 is involved in the inflammatory response via some mediators, including TREM-1	COX-2-siRNA	Monocytes/macrophages, lymphocytes and mast cells	*in vitro*
Yu Du *et al*. ^26^	Nrf2	FLS increased the level of reactive intracellular oxygen species via TNF-*α*	Nrf2-siRNA	FLS	*in vitro*
Choi *et al*. ^28^	EGR3	EGR3 promotes Cyr61-induced cell migration and invasion	EGR3-siRNA	FLS	*in vitro*
Wakabayashi *et al*. ^29^	CCL11	CCL11 induces the migration of different leukocyte types by interacting with CCR3	CCL11-siRNA	FLS, monocytes	*in vitro*
Xu *et al*. ^30^	E2F2	E2F is involved in cell proliferation and survival, cell signaling and cell cycle regulation	E2F2-siRNA	FLS	*in vitro*
Wang *et al*. ^27^	SphK1	SphK1 is involved in the angiogenesis process via VEGF	SphK1-siRNA	FLS	*in vitro*
Saruga *et al*. ^15^	MDA5	MDA5 is an RNA helicase that plays a role in innate immune and inflammatory reactions	MDA5-siRNA	FLS	*in vitro*
Zhao *et al*. ^32^	RANKL	RANKL is the most important inducer of osteoclastogenesis and is expressed and upregulated in the synovial tissues of the RA	RANKL-siRNA	Synovial cells	*in vitro*
Yoon and Moon^31^	hBAFF	hBAFF regulates the maturation, maintenance and apoptosis of B cells	hBAFF-siRNA	FLS	*in vitro*

^*^HIF-1*α*, Hypoxia-inducible factor-1*α*; Sp1, Specificity protein 1; COX-2, Cyclooxygenase-2; PRMT5, Protein arginine methyltransferase 5; HMGB1, High-mobility group box 1; TS, Thymidylate synthase; Cyr61, Cysteine-rich protein 61; Mcl-1, Myeloid cell leukemia-1; RRM2, Ribonucleotide reductase M2; STAT3, Signal transducer and activator of transcription 3; PDK-1, Phosphoinositide-dependent kinase-1; Nrf2, Nuclear factor erythroid 2-related factor 2; EGR3, Early growth response 3; CCL11, Chemokine C–C motif ligand 11; E2F2, E2F transcription factor 2; SphK1, Sphingosine kinase-1; MDA5, Melanoma differentiation-associated gene 5; RANKL, Receptor activator of nuclear factor *κ*B ligand; hBAFF, B cell-activating factor.

Of these studies, 15 used siRNAs to silence specific genes and then identify gene and protein targets to produce targeted therapy. Another five studies used siRNAs to monitor the function of some RA drugs.

No scientific studies were identified on human cells and siRNA on the topic of RA before 2012.

Lee *et al.* evaluated hypoxia in the pathogenesis of RA. They cultured two groups of fibroblasts, the first under hypoxic conditions and the second in normoxic conditions under stimulation with interleukin 1 beta (IL-1*β*). The inflammatory response was assessed by measuring the values of vascular endothelial growth factor (VEGF), metalloproteinase (MMP)-1 and MMP-13 in the two cell groups.^21^ The group of fibroblasts in hypoxic conditions was transfected with siRNA specific for hypoxia-inducible factor-1*α* (HIF-1*α*). The differential expression of MMP under the combined effect of IL-1*β* and hypoxia was significantly attenuated by silencing HIF-1*α* with siRNA.^21^

Small interfering RNAs have been used to determine the effect of some proteins in regulating the inflammatory processes in RA, to identify these proteins and their possible use as a therapeutic target. In this regard, Chen *et al.*, studying the enzyme arginine methyltransferase 5 (PRMT5) found that this enzyme increases in fibroblast-like synoviocytes (FLS) from patients with RA. In siRNA-treated cells, they demonstrated reduced production of IL-6 and IL-8 and proliferation of RA FLS.^22^

Xu *et al*. analyzed cysteine-rich protein 61 (Cyr61), a product of an immediately early gene. Cyr61 is directly correlated with adhesion, cell migration and stimulation of production of specific inflammatory cytokines. Experimentally, Cyr61-siRNA reduced matrix MMP-3 and MMP-13 levels and induced apoptosis in RA FLS cells. In addition, a specific monoclonal antibody against Cyr61 showed good results in reducing inflammation in RA in mice. Based on the results obtained, the authors began testing a similar monoclonal antibody on humans.^23^

To silence a specific gene, siRNAs must be delivered effectively. Nogueira *et al*. evaluated the efficiency of folate-targeted liposomes for specific delivery of siRNA to activated macrophages, key effector cells in RA pathology that specifically express the folate receptor*β*. They incorporated human liposomes with siRNA-myeloid cell leukemia-1 (Mcl-1).

Myeloid cell leukemia-1 is a protein essential for the survival of synovial macrophages.

The liposomal formulation used by Nogueira *et al*. was effective in the transport of siRNA-Mcl-1, inhibiting Mcl-1 expression in treated human macrophages.^24^

An additional gene target for RA therapy has been proposed by Wang *et al*. They developed a peptide–polycation lipid-protamine-DNA (LPD) conjugated cell-permeable complex loaded with ribonucleotide reductase M2 (RRM2) siRNA (CCP-LPDR), aiming to increase apoptosis levels and inhibit proliferation of RA FLS. CCP-LPDR is a small molecule (~130 nm) with a high siRNA encapsulation efficiency ( >90%) and high stability.^25^ This resulted in approximately 80% suppression of RRM2 gene and protein expression. In particular, CCP-LPDR resulted in a marked decrease in proliferation and an increase in the level of apoptosis in RA FLS. In addition, the levels of proinflammatory cytokines, tumor necrosis factor alfa (TNF-*α*) and IL-6, were decreased in RA FLS after treatment with CCP-LPDR.^25^

A much better-known mediator was studied by Peng *et al*., who inhibited cyclooxygenase-2 (COX-2) by a specific siRNA, responsible for the inflammatory response and particularly active in RA.

COX-2 is involved in the inflammatory response via some mediators, including triggering receptor expressed on myeloid cells-1 (TREM-1).

TREM-1 is directly connected and overexpressed by the cytokines produced through COX-2, and determines the activation of the inflammatory response by acting on monocytes/macrophages, lymphocytes and mast cells. TREM-1 may be a good therapeutic target in human RA.^14^

Silencing of melanoma differentiation-associated gene 5 and nuclear factor erythroid 2 by specific siRNAs demonstrated a reduction in the inflammatory process in FLS *in vitro*.^15^^,^^26^

Wang *et al*. instead studied the effect of VEGF in maintaining the inflammatory process in RA.^27^

Other authors have used siRNA in cell cycle regulation, migration and cell apoptosis in both B and inflammatory RA cells.^28–31^

The biological mechanism of siRNAs has been used to identify the target proteins and genes^14^^,^^15^^,^^17^^,^^21–32^ of some already existing drugs,^16^^,^^17^^,^^19^^,^^33^^,^^34^ to test their efficacy in RA.

Ikuta *et al*. analyzed the specificity protein 1 (Sp1) protein that binds the promoter of the gliostatin gene (GLS). Gliostatin/thymidine phosphorylase has angiogenic and arthritogenic activities, and aberrant GLS production has been observed in the active synovial membranes of RA patients. The use of the drug mithramycin reduced the expression of Sp1 and GLS in RA FLS, thus defining mithramycin as a useful RA drug.^16^

Some authors have experimentally used a therapeutic combination (siRNA-COX-2 and dexamethasone). The two drugs were inserted into poly (DL-lactic-co-glycolic acid) nanoparticles that directed them into target cells (immortalized chondrocyte cell line C-28/I2). The result was apoptosis of 30% of the cells analyzed and the reduction of COX-2 expression. The combined therapy was shown useful in the treatment of RA.^17^

Li *et al*. conducted a twofold study. They analyzed the effects of siRNA-high-mobility group box 1 (HMGB1) on FLS and studied the therapeutic effects of methotrexate (MTX) in RA. HMGB1 is actually overexpressed in RA patients, and its activity is reduced by using siRNA-HMGB1. This effect is evident in the reduction of matrix MMP-2 and MMP-13 present in the RA. Methotrexate in turn resulted in a reduction in HMGB1, thus showing itself to be a good anti-RA drug.^18^

Municio *et al*. also studied the therapeutic effects of MTX. Their study was conducted on granulocyte-macrophage colony-stimulating factor (GM-CSF) or macrophage CSF polarized macrophages. To analyze the molecular pathways stimulated in the RA process, and therefore the function of MTX, they used specific siRNA. Thymidylate synthase (TS) and protein 53 (p53) were expressed by polarized cluster of differentiation (CD)163 +/TNF-α + GM-CSF macrophages from RA joints but not normal synovium. The macrophage response to MTX is polarization-dependent and determined by the TS-p53 axis.^19^

Li *et al*. investigated the immunological mechanisms by which arsenic trioxide (As2O3) can inhibit the differentiation of T helper 17 (Th17) cells by promoting the generation of regulatory T (Treg) cells by modulating the signal transducer and activator of transcription 3 (STAT3) in treatment-naïve RA patients. To test the mechanism of As2O3 on Th17–Treg balance *in vitro*, they conducted STAT3 transfection experiments with siRNAs. Arsenic trioxide may be a potential immunomodulator for treatment-naïve RA patients that helps balance Treg and Th17 cells through modulation of STAT3.^33^

Ma *et al*. compared the action of the antimalarial drug artesunate by comparing it to two other drugs, hydroxychloroquine and MTX, using FLS from RA patients. Biological pathways were investigated and tested by phosphoinositide-dependent kinase-1 (PDK-1) knockdown by siRNA transfection. Artesunate inhibited RA FLS migration and invasion through the suppression of PDK1-induced activation of protein kinase B and ribosomal S6 kinase 2phosphorylation. Artesunate exerted greater anti-inflammatory effects than hydroxychloroquine and similar to MTX. *In vitro*, MTX and artesunate also had a synergistic effect on the inhibition of RA processes. Artesunate may be a potential disease-modifying antirheumatic drug for RA.^34^

## Discussion

RA is the most severe condition in terms of structural damage to the joints, secondary bone damage, extra-articular complications, associated comorbidities and risk of mortality.^3^ In RA, as with other autoimmune diseases, the immune system attacks healthy tissues, not recognizing them as such. The preferred target of antibodies in RA is the synovial membrane. The membrane reacts to such proinflammatory stimuli by increasing in volume and giving rise to abundant synovial tissue. This expands to cause gradual destruction of articular cartilage, but the proliferative process in severe cases reaches the bones and other surrounding tissues, including subchondral bone, capsules, tendons and ligaments. RA produces marked disability.^2^

The cause of RA is not yet fully known, but its pathogenesis is multifactorial.^2^

Our current study focuses on possible therapies and the possibility of using siRNAs as therapy or in the experimentation of new drugs.

Authors have carried out studies on human cells, FLS, chondrocyte, monocytes/macrophages, lymphocytes and mast cells. The aim was to highlight the organic pathways and the molecular targets on which it is possible to intervene.

All cell types, and mainly fibroblasts, overexpress IL-1, TNF-*α* and TNF-*β* in patients with AR.^35^

TNF-*α*, a cytokine involved in systemic inflammation, is a member of a group of cytokines that stimulates the reaction of the acute phase. It is produced by T-CD4+ lymphocytes, natural killer (NK) cells, neutrophiles, mast cells, eosinophilic and neurons. TNF-*α* is the main cell regulator of the immune system.^36^

Proinflammatory cytokines can influence VEGF and MMP gene expression in synoviocytes during progressive inflammation. In RA, joint inflammation and the production of proinflammatory cytokines, including IL-1 and TNF-*α*, stimulate synovial fibroblasts to produce large amounts of MMP.^35^^,^^37^

The expression of MMP is significantly reduced when TNF-*α* blocking agents are used.^38^^,^^39^

There are about 20 different members of the MMP family.^40^^,^^41^ Of these, MMP-1 and MMP-13 seem to be the most important in the degradation of cartilage in RA because they limit the rate of collagen degradation.^42^^,^^43^ MMP-1 and MMP-13 are the only ones able to split the native triple helix of collagen, allowing it to unwind from the chains; after unwinding, the chains may undergo further degradation by other MMPs.

In animal models, inhibitors with varying degrees of selectivity for different MMPs showed cartilage protective activity in AR.^44^ However, the development of MMP inhibitors as therapeutic agents in clinical trials has been hindered by side effects.^45^^,^^46^ Therefore, the factors influencing individual MMP expression require further clarification to allow efficient control of MMP expression in clinical treatments.

IL-1*β* and TNF-*α* are proinflammatory cytokines that play an important role in RA. They denature the associated cartilage matrix through the production of MMP, and induce the consequent reduction in the production of proteoglycans and collagen.^47^^,^^48^ IL-1*β* and TNF-*α* promote the expression of the inflammation-related genes COX-2 and inducible nitric oxide synthase,^36^^,^^49^ resulting in high levels of prostaglandin E2 and nitric oxide production, respectively.^50^^,^^51^

COX-2 is both inducible and constitutive (in the central nervous system, kidneys and intestines).

Specific stimulatory events activate and regulate COX-2, which, in turn, activates the biosynthesis of prostanoids involved in inflammation.^52^

COX-2 expression is upregulated in autoimmune diseases such as RA and in many neoplasms.^53^

*In vivo* studies have shown the effectiveness of using siRNA in pathologies other than AR.^54^^,^^55^

Jyotsana *et al.* have shown that RNAi therapies can be used against leukemia cells and promise new treatment options for leukemia patients.^55^

## Conclusion

The reguardies who study autoimmune diseases aim to find a highly selective therapy that only acts on the pathological inflammatory process. Numerous drugs have been proposed in RA but none is entirely specific. The purpose of using siRNAs is 2-fold. On the one hand, they can be used to accurately determine the genes and molecules involved in RA; on the other, they can be used as a target therapy.

Numerous drugs have been tested in animal cells. We analyzed the results obtained with siRNAs in human cell therapy. *In vitro*, the results are satisfactory, demonstrating their possible application *in vivo*. Gene therapy is the future for many pathologies, and its application may be useful in RA, a condition in which we do not know the causes but we know the molecular targets on which we can act.

## Data availability

Any data are reported.
